# The tumor suppressive miR-200b subfamily is an ERG target gene in human prostate tumors

**DOI:** 10.18632/oncotarget.9366

**Published:** 2016-05-13

**Authors:** Zheng Zhang, Rainer B Lanz, Lijuan Xiao, Lei Wang, Sean M Hartig, Michael M Ittmann, Qin Feng, Bin He

**Affiliations:** ^1^ Department of Molecular and Cellular Biology, Baylor College of Medicine, Houston, Texas, USA; ^2^ Department of Medicine-Hematology and Oncology, Baylor College of Medicine, Houston, Texas, USA; ^3^ Department of Pathology and Immunology, Baylor College of Medicine, Houston, Texas, USA

**Keywords:** prostate cancer, ERG, miR-200, miR-205

## Abstract

The TMPRSS2-ERG fusion occurs in approximately 50% of prostate cancer (PCa), resulting in expression of the oncogenic ERG in the prostate. Because ERG is a transcriptional activator, we hypothesized that ERG-regulated genes contribute to PCa development. Since microRNA (miRNA) has crucial functions in cancer, we searched for miRNAs regulated by ERG in PCas. We mined published datasets based on the MSKCC Prostate Oncogene Project, in which a comprehensive analysis defined the miRNA transcriptomes in 113 PCas. We retrieved the miRNA expression datasets, and identified miRNAs differentially expressed between ERG-positive and ERG-negative samples. Out of 369 miRNAs, miR-200a, −200b, −429 and −205 are the only miRNAs significantly increased in ERG-positive tumors. Strikingly, miR-200a, −200b and −429 are transcribed as a single polycistronic transcript, suggesting they are regulated at the transcriptional level. With ChIP-qPCR and *in vitro* binding assay, we identified two functional ETS motifs in the miR-200b/a/429 gene promoter. Knockdown of ERG in PCa cells reduced expression of these three miRNAs. In agreement with the well-established tumor suppressor function, overexpression of the miR-200b/a/429 gene inhibited PCa cell growth and invasion. In summary, our study reveals that miR-200b/a/429 is an ERG target gene, which implicates an important role in TMPRSS2/ERG-dependent PCa development. Although induction of the tumor suppressive miR-200b subfamily by oncogenic ERG appears to be counterintuitive, it is consistent with the observation that the vast majority of primary prostate cancers are slow-growing and indolent.

## INTRODUCTION

miRNAs are small non-coding endogenous RNAs of approximately 22 nucleotides that regulate gene expression by directing their target mRNAs for degradation and/or translational repression. miRNAs are pleiotropic modulators in all important biological processes, including development, differentiation, immunity, heart disease, and cancer. In cancer, miRNAs can regulate tumor initiation and progression, and factors involved in miRNA biogenesis are frequently dysregulated. Certain miRNAs function as oncogenes (oncomiR). For instance, miR-21 is overexpressed in most tumor types [1] where it downregulates the tumor suppressor PDCD4 [2, 3]. The oncogenic miR-17-99 cluster is located at 13q31, which is frequently amplified in solid tumors and in lymphomas [4, 5]. The expression of the miR-17-99 cluster is induced by oncogenic c-Myc [6]. miR-155 is another oncomiR highly expressed in Hodgkin's lymphomas and B cell lymphomas [7, 8]. miRNAs can also function as tumor suppressors [9, 10]. The miR-15a/16-1 cluster at 13q14.3 is frequently deleted in B-cell chronic lymphocytic leukemia (B-CLL) [10], and miR-15a and miR-16-1 are bona fide tumor suppressors, which act by targeting oncogene Bcl-2 [11]. The miR-34 family, which consists of miR-34a, b and c, is induced by p53 and functions as a key regulator of tumor suppression [12–16]. The Let-7 family of miRNAs inhibits tumor growth and metastasis by targeting the oncogenes H-Ras, HMGA2 and c-Myc [17–19].

Another family of well-established tumor suppressive miRNAs is miR-200, consisting of miR-200a, −200b, −200c, −141, and −429, which are clustered at two genomic locations. In the human genome, miR-200b, −200a, and −429 are located on chromosome 1 and transcribed by RNA Polymerase II as a single long primary miRNA transcript [20], while miR-200c and −141 are clustered on chromosome 12. The miR-200 family inhibits cancer cell invasion and metastasis by suppressing epithelial-mesenchymal transition through targeting ZEB1 and ZEB2 [21–24]. Genome-wide analysis of miR-200 targets in living cells has confirmed that miR-200 prevents cell migration and invasion through a coordinate control of actin cytoskeleton dynamics [25]. In addition, miR-200 family members can block tumor angiogenesis by targeting interleukin-8 and CXCL1 [26]. In agreement with its tumor suppressor role, the expression of miR-200 family members has been associated with favorable clinical outcome in many cancers [27–29].

In contrast to other well-characterized oncomiRs and tumor suppressors, the role of some miRNAs in cancer is controversial and context-dependent. For instance, miR-205 is highly enriched in progenitor cells in the mammary gland and prostate, and has a function in stem cell maintenance [30–32]. miR-205 is oncogenic in the mammary gland by targeting the tumor suppressor PTEN [30]. On the other hand, miR-205 can also inhibit tumor invasion by targeting ZEB1 and ZEB2 [21]. miR-205 expression levels were significantly lower in prostate cancer than in normal prostate, suggesting a tumor suppressor function [33]. However, in normal prostate, miR-205 is preferentially expressed in basal epithelial cells [31]. As a result, reduced miR-205 expression in human prostate cancer is probably caused by the loss of basal cells, instead of being lost in luminal cells as a tumor suppressor.

In prostate cancers, TMPRSS2-ERG fusion caused by chromosomal translocation is present in approximately 50% of samples and manifests over-expression of a functional ERG transcription factor [34]. ERG modulates AR signaling in VCaP cells [35, 36] and the murine prostate gland [37], and therefore contributes to prostate cancer progression. TMPRSS2/ERG also cooperates with Pten loss to promote prostate oncogenesis in mouse models [38, 39]. However, how ERG controls prostate tumorigenesis and progression remains largely unknown.

ERG is a member of the erythroblast transformation-specific (ETS) family of transcription factors. It contains a highly conserved DNA binding ETS domain, which binds to DNA elements with a central GGAA motif. We reasoned that, as a transcriptional activator, ERG protein *per se* might not directly control cancer development. Instead, the downstream target genes regulated by ERG could determine prostate cancer initiation and progression. In a search for ERG target genes, we used recently published genomic profiling of human prostate cancer [40], in which a comprehensive analysis was applied to define miRNA transcriptomes in 113 prostate tumors. We retrieved the miRNA expression data sets, divided them based on ERG expression levels, and looked for miRNAs differentially expressed between ERG-positive and ERG-negative samples. Strikingly we found that miR-200a, b, −429, and −205 are the only four miRNAs significantly increased in ERG-positive tumors. In this study, we provide definitive evidence that the miR-200b/a/429 subfamily is an ERG target gene in human prostate cancers.

## RESULTS

### Identification of ERG-associated miRNAs in human prostate cancers

**Figure 1:**
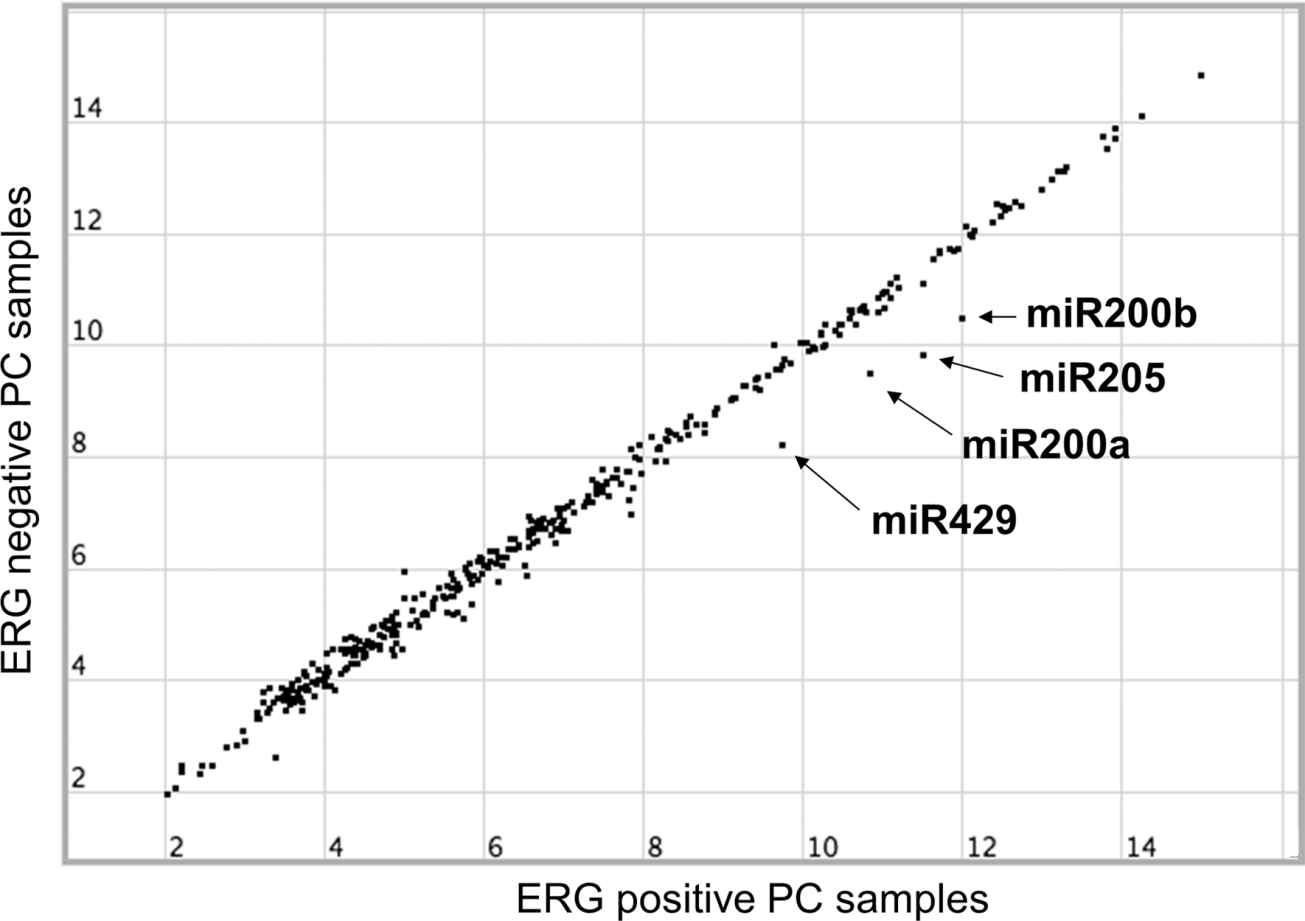
Scatter representation of miRNA expression levels grouped by the patient's ERG mRNA expression Average expression levels of 369 microRNAs plotted for their relationship between ERG-positive (40 patients) and ERG-negative (60 patients) expression, after removal of 13 samples with moderate ERG levels. Log2 values of average expression for ERG positive/negative PCa and *p*-value for the four miRNAs that are the focus of this study are: miR-200a (10.85/9.49, *p* = 1.88e-05), miR-200b (11.99/10.51, *p* = 1.42e-06), miR-205 (11.50/9.84, *p* = 9.85e-04), miR-429 (9.73/8.22, *p* = 1.59e-05). miRNA and ERG expression data are from the Memorial Sloan-kettering Cancer Center, New York [40].

Because ERG is a transcription activator and miRNAs are emerging as crucial regulators of cancer development, we tested the hypothesis that ERG directly regulates miRNA expression in human prostate cancers. We retrieved published miRNA expression data sets [40], and based on ERG expression levels, we classified the tumor samples into ERG-positive and ER-negative groups. We then looked for miRNAs differentially expressed between ERG-positive and ERG-negative samples. As shown in Figure 1, out of 369 miRNAs, the average expression levels of four miRNAs including miR-200a, miR-200b, miR-429, and miR-205 were significantly higher in ERG-positive human prostate cancer samples. Strikingly, miR-200a, miR-200b and miR-429 are known to be transcribed as a single polycistronic transcript, suggesting that these three miRNAs are regulated by ERG at the transcriptional level.

### Identification of ERG binding motifs in the promoter region of miR-200b/a/429 gene cluster

Strong positive association of these four miRNAs with ERG expression suggests that these miRNAs might be directly regulated by ERG at the transcriptional level in human prostate cancers. By taking advantage of published ERG chromatin immunoprecipitation (ChIP-Seq) data sets, we investigated if ERG directly binds to the regulatory regions near the miR-200b/a/429 cluster and miR-205 gene in prostate cancer cells.

**Figure 2:**
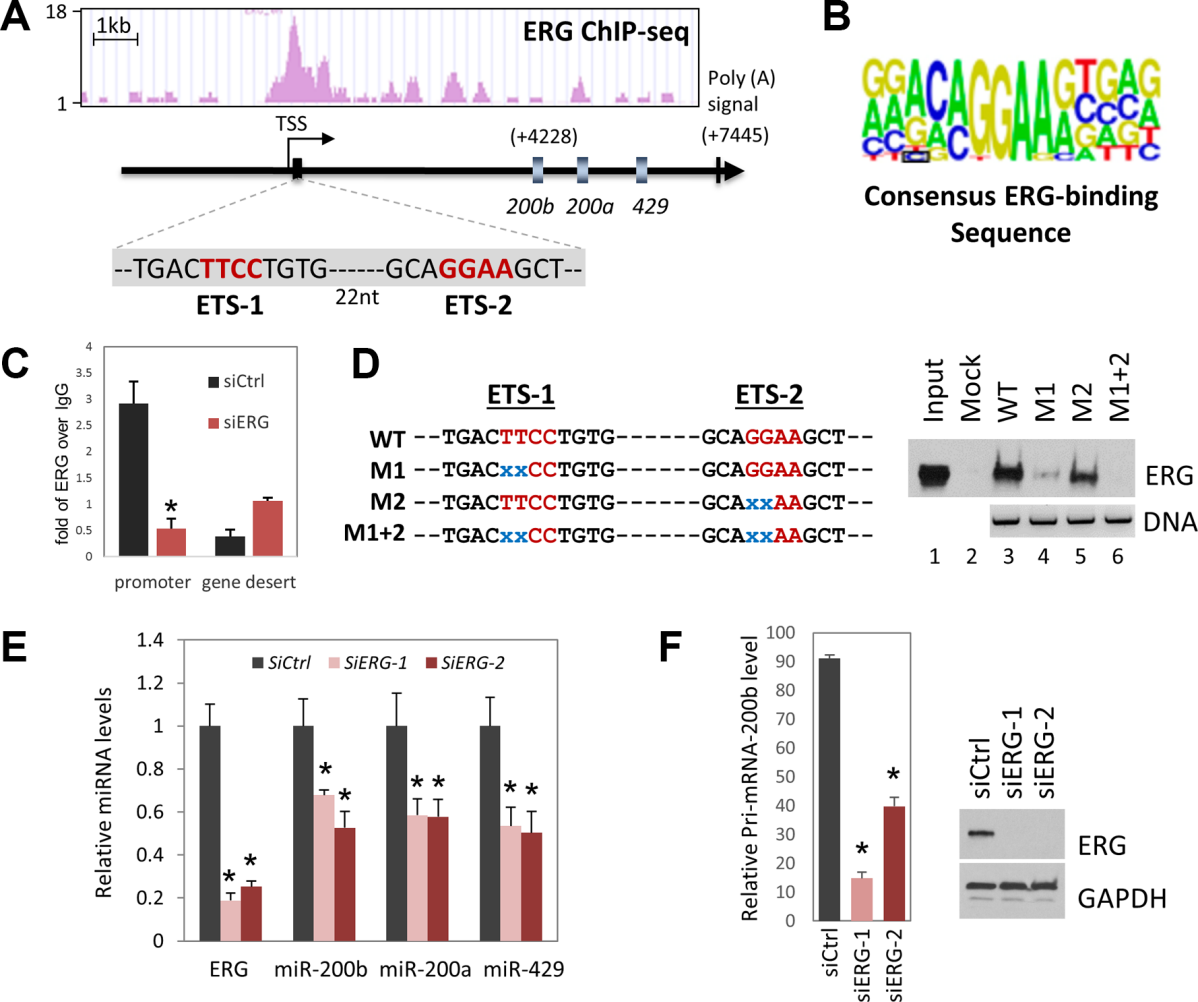
Regulation of miR-200b/200a/429 cluster gene expression by ERG at the transcriptional level (**A**) ERG ChIP-seq data sets were retrieved, including GSM717395, GSM717397, GSM717392, and GSM717394. ERG binding peaks were viewed by UCSC genome browser. *In silico* analysis reveals two classical ERG binding elements (Ets) in the promoter region. TSS, transcription start site. (**B**) Diagram of consensus ERG-binding sequence derived from genome-wide ChIP-seq analyses. (**C**) Confirmation of ERG binding to the promoter of miR-200b/a/429 gene by individual ChIP-qPCR analysis. VCaP cells were treated with negative control siRNA or ERG siRNA for three days before being fixed for ChIP assay. The purified ChIP DNA was used in qPCR to amplify a region in a gene desert (Active Motif, cat No. 71001) and miR-200b promoter region. Data are presented as fold changes of ERG enrichment over IgG enrichment. (**D**) *In vitro* binding of ERG protein to wild type, but not mutant miR-200b/a/429 promoter region. “X” in blue stands for deletion of the corresponding nucleotide. (**E**) Reduced miR-200b, miR-200a, and miR-429 expression levels in VCaP cells after being treated with two individual ERG siRNAs (stealth siRNA, Invitrogen) for four days. Error bars, mean ± SEM. *P < 0.05. (**F**) Reduced miR-200b/a/429 primary transcript levels in VCaP cells after being treated with ERG siRNA for three days. Error bars, mean ± SEM. *P < 0.05. Western blot analysis was performed to confirm efficient ERG knockdown.

Among the commonly used prostate cancer cell lines, VCaP is the only cell line that harbors TMPRSS2/ERG translocation and expresses ERG protein. Two research groups have published genome-wide ERG ChIP-seq analyses in VCaP cells [35, 36]. We retrieved the data sets from both studies and examined the ERG sites in the mir-200b/a/429 gene cluster and miR-205HG gene using the UCSC genome browser. Shown in Figure 2A, based on ERG ChIP-seq results [36], we nominated an ERG binding peak immediately proximal to the transcription start site (TSS) of mir-200b/a/429 gene cluster (Figure 2A). This ERG binding peak was confirmed by the other ChIP-seq analysis [35] (data not shown). On the other hand, expression of miR-205 is transcribed from its host gene miR-205HG on chromosome 1. Surprisingly, no ERG binding peak was identified within 25 kbp upstream or downstream of the miR-205HG gene.

Within the ERG binding site in the miR-200b/200a/429 gene promoter, we identified two potential ERG binding sequences, ETS-1 and ETS-2 (Figure 2A), that match the canonical ERG binding sequences derived from genome-wide ERG ChIP-seq analyses [41] (Figure 2B). Next we confirmed that ERG indeed binds to this site in VCaP cells by ChIP-qPCR analysis (Figure 2C). To further determine whether these two potential binding sequences ETS-1 and ETS-2 are bona fide ERG binding motifs, we performed an *in vitro* binding assay. Shown in Figure 2D, 168 bp of miR-200b promoter region containing ETS-1 and ETS-2 was amplified by PCR with biotinylated primers. ETS-1 and ETS-2 were mutated individually or in combination: WT, two both sites intact; M1, ETS-1 mutated; M2, ETS-2 mutated; M1+2, both sites mutated. These DNA fragments were bound to streptavidin agarose beads, and incubated with VCaP total cell lysates, which contain the endogenous ERG proteins. After binding at 4°C, the agarose beads were precipitated, washed, and bound proteins were denatured and separated by SDS-PAGE gel. The presence of ERG was detected by western blot analysis using anti-ERG antibody. In the right panel of Figure 2D, wild type sequence shows robust ERG protein binding (lane 3). Mutation of ETS-1 dramatically reduced the binding (lane 4) and mutation of ETS-2 moderately reduced the binding (lane 5), while mutation of both sites completely abolished binding (lane 6). This *in vitro* binding assay demonstrates that the promoter region of miR-200b/200a/429 contains two functional ERG binding motifs which recruit ERG efficiently.

Next, in order to test whether expression levels of miR-200a, miR-200a, miR-429 are regulated by ERG in VCaP cells, we used siRNA to specifically knock down ERG and used qPCR to measure mature miRNA levels. As shown in Figure 2E, treatment of VCaP cells with two individual ERG siRNAs (Stealth siRNA, Invitrogen) for four days caused approximately 40% reduction in the expression levels of miR-200b, miR-200a, and miR-429. The expression level of miR-200b/a/429 primary transcript was also significantly reduced after VCaP cells were treated with ERG siRNA for three days (Figure 2F). Taken together, our results indicate that miR-200b, miR-200a, and miR-429 are directly and positively regulated by ERG at the transcriptional level in VCaP cells. The remaining expression of miR-200b subfamily in VCaP cells after ERG knockdown is in agreement with a previous report that SP1 controls the basal epithelial expression of miR-200b subfamily [20]. On the other hand, expression of miR-205 was undetectable in VCaP cells by qPCR. Therefore, we cannot determine whether miR-205 is regulated by ERG in prostate cancer cells.

### Effects of miR-200b/a/429 cluster and miR-205 on prostate cancer growth and migration

**Figure 3:**
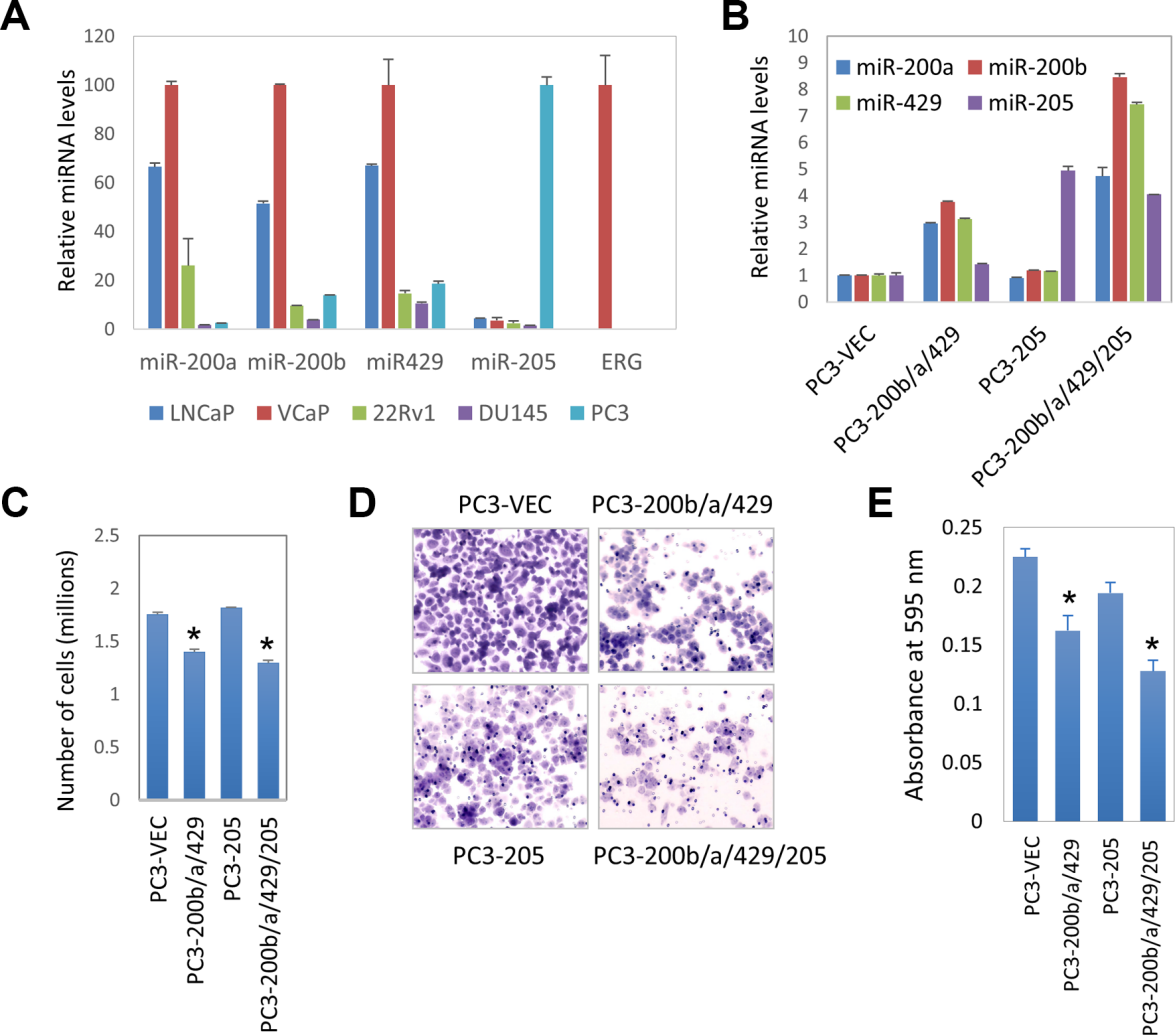
Role of miR-200b subfamily and miR-205 on prostate cancer proliferation and invasion (**A**) Expression levels of miR-200a, miR-200b, miR-429, miR-205, and ERG in commonly used prostate cancer cell lines, including LNCaP, VCaP, 22RV1, PC3, DU145. Mature miRNAs expression levels were measured by qRT-PCR, normalized against RNU48 internal control. (**B**) Expression of miRNAs in PC3 stable cell lines, including PC3-vec, PC3-miR-200b/a/429, PC3-miR-205, and PC3-miR-200b/a/429/205. (**C**) The effect of miRNAs on PC3 cell growth as determined by MTT assay. (**D**) PC3 stable cell Matrigel invasion assay using BD Biocoat Matrigel Invasion Chambers. Representative pictures of invaded cells stained with crystal violet. Four stable cell lines, PC3-vec, PC3-miR-200b/a/429, PC3-miR-205, and PC3-miR-200b/a/429/205. (**E**) Quantification of invasion. Invaded cells were stained with crystal violet, which was then solubilized in 1% SDS. The absorbance was measured at 595 nm. Error bars, mean ± SEM. *P < 0.05.

The roles of miRNA-200 family and miR-205 have been studied in many cancer types. However, their roles in human prostate cancers are relatively unknown. We first measured the expression levels of these miRNAs in commonly used human prostate cancer cell lines by quantitative real-time PCR (qRT-PCR), including LNCaP, VCaP, 22RV1, DU145, and PC3 cells. As shown in Figure 3A, miR-200b, miR-200a, miR-429 are highly expressed in VCaP and LNCaP cells, moderately expressed in 22RV1 cells, and weakly expressed in DU145 and PC3 cells. As a control, ERG mRNA is only abundantly expressed in VCaP cells. In contrast, miR-205 was highly expressed in PC3, but weakly expressed in other cell lines. In normal murine prostate, miR-205 is preferentially expressed in basal epithelial cells, but not in luminal epithelial cells [31]. Expression of miR-205 in PC3 cells suggests that PC3 cells might have acquired certain basal/progenitor cell characteristics. Interestingly, ERG is not expressed in PC3 cells, suggesting that miR-205 expression in prostate cancer cell line can be ERG-independent.

Next, we tested the functions of miR-200b/a/429 and miR-205 in human prostate cancer cell lines. Using lentivirus, we generated stable cell lines that express miR-200b/a/429 cluster and/or miR-205 in PC3 cells. In Figure 3B, the expression levels of these four miRNAs in the PC3 stable cell lines were as anticipated. Shown in Figure 3C, overexpression of miR-200b/a/429 gene cluster alone, or in combination with miR-205, significantly inhibited the cell proliferation rate as determined by the MTT assay. However, overexpression of miR-205 alone did not inhibit the cell proliferation.

We then investigated the effect of these miRNAs on cancer cell invasion using an *in vitro* Matrigel Invasion assay. Shown in Figure 3D and 3E, overexpression of miR-200b/a/429 cluster dramatically inhibited the invasion, while overexpression of miR-205 was only moderately inhibitory. Overexpression of four miRNAs simultaneously had the most significant inhibitory effect on cell invasion. Our results are in agreement with numerous previous reports that the miR-200 family members inhibit cancer cell invasion in many cancer types.

### These miRNAs are not induced by ERG in murine prostate in TMPRSS2/ERG transgenic mice

**Figure 4:**
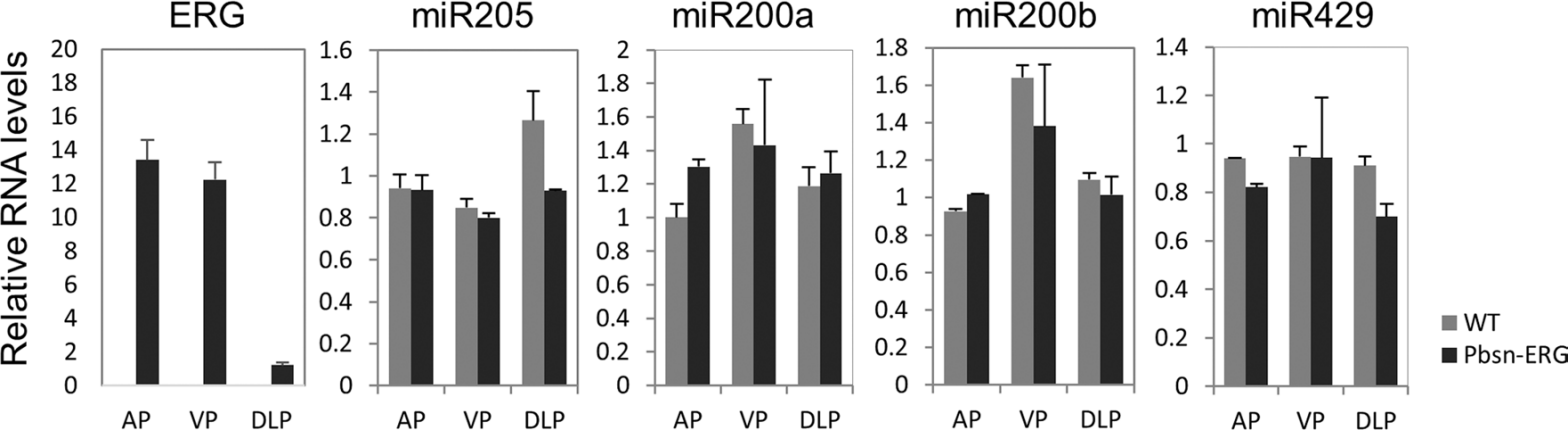
miR-200b/200a/429 subfamily and miR-205 are not induced by ERG in the prostate of Pbsn-ERG transgenic mice Anterior (AP), dorsal-lateral (DLP), and ventral (VP) prostate lobes were harvested from wild type litter mates (*n* = 3) and Pb-ERG transgenic mice (*n* = 2). Real-time qPCR was used to determine expression levels of human ERG, and mouse miR-200b, miR-200a, miR-429 and miR-205. Error bars, mean ± SEM.

Although TMPRSS2/ERG fusion is detected in approximately 50% of human prostate tumors, ERG transgenic mice do not develop prostate tumor spontaneously [37]. One explanation is that downstream target genes regulated by ERG in human prostate are not induced by ERG in murine prostate due to lack of conservation between human and mouse genomic sequences. We obtained ERG transgenic mice from the Jackson Laboratory [42], and dissected the prostate lobes, including anterior, ventral, and dorsal-lateral lobes. In Figure 4, ERG expression was robust in all the lobes in ERG transgenic mice, but not in wild type control litter mates. However, the expression levels of miR-200b, miR-200a, miR-429, and miR-205 in transgenic mice are similar to that of wild type litter mates, indicating that ERG cannot induce the expression of these four miRNAs in murine prostate. This is probably due to the lack of ERG binding motifs in the promoter region of murine mir-200b/a/429 gene (data not shown).

## DISCUSSION

In this study, we found that, out of 369 miRNAs, the expression levels of four miRNAs, including three members of the miR-200b subfamily and miR-205, are positively associated with ERG expression in MSKCC prostate cancer data sets. Interestingly, in a previous report, miR-200c is found to be an ERG target gene in VCaP cells and miR-200c expression is actually negatively regulated by ERG [43, 44]. Although there is no association between miR-200c and ERG expression levels in the MSKCC cohort of human prostate tumor samples based on our analysis, we cannot rule out the possibility that miR-200c expression might be inversely correlated with ERG expression in different cohorts of human prostate cancer samples and thus has a role in TMPRSS2/ERG-dependent prostate cancer development.

A quite surprising finding is that miR-205 expression is positively correlated with ERG, however, the underlying mechanism is unknown. ChIP-seq analyses reveal no ERG binding site near the miR-205 host gene (+/− 25 kbp) in VCaP cells. We cannot rule out the possibility that ERG might regulate miR-205HG transcription by a long distance looping mechanism, or a transcription factor regulated by ERG regulates miR-205 expression. However, the expression of ERG and miR-205 is mutually exclusive in cultured prostate cancer cells (Figure 3A). Additionally in prostate tissue, miR-205 is regulated by TP63 [45] and highly enriched in the basal epithelial cells of the prostate [31], while TMPRSS2 promoter-driven ERG is expressed in the luminal epithelial cells. Therefore miR-205 and ERG are expressed in different cell compartments in the prostate. The mutual exclusive expression pattern suggests that association of ERG and miR-205 in human prostate cancers might be through an indirect mechanism. Because basal epithelial cells are lost during prostate cancer development, it is possible that TMPRSS2/ERG-dependent prostate tumors retain more basal cells, resulting in high levels of miR-205 in the tumors. This possibility remains to be investigated.

One exciting finding of our study is that ERG directly up-regulates the tumor suppressive miR-200b subfamily of miRNAs in prostate cancer. Although the miR-200 family of miRNAs can also be regulated at post-transcriptional levels [46], our evidence indicate that ERG can directly bind to miR-200b/a/429 promoter to facilitate its transcription in prostate cancer cells. High levels of miR-200 predict favorable survival in many cancers [27–29]. This observation immediately raises an interesting question: Is ERG a predictive biomarker for favorable clinical outcome? In support of this speculation, previous studies report that TMPRSS2/ERG fusion is indeed a predictor of favorable outcome for prostate cancer patients [47, 48]. On the contrary, many studies suggest that TMPRSS2/ERG is a predictor for poor clinical outcome [49–51]. There are also reports indicating that ERG status is not predictive for prostate cancer recurrence or progression after radical prostatectomy [52, 53].

Such controversy on the prognostic value of ERG in prostate cancer might be due to the following reasons. First, it is possible that the miR-200b subfamily has tumor stage-dependent activity (53). Previous reports show that miR-200 could promote breast cancer metastatic colonization by targeting Sec23a [54]. As a result, the activity of enhancing metastatic colonization by miR-200b at late stage may offset its anti-invasion activity at early stage. The second possibility is that ERG might also regulate the expression of protein-coding genes in prostate cancer, in addition to miR-200b subfamily. These protein-coding target genes might increase the risk of cancer recurrence and progression and therefore offset the beneficial effect of the miR-200b subfamily. Thirdly, the TMPRSS2/ERG fusion prevalence is significantly different in prostate cancers from different ethnic groups [53]. In comparison to Caucasian patients, ERG expression is much less common in prostate cancers in African American [55, 56] and Asian populations [57]. It is possible that in different ethnic groups, ERG may have different prognostic value. For example, in African American patients, ERG-negative status is found to be associated with high-grade cancers [58]. More studies are required to determine the prognostic value of ERG in different ethnic groups, particularly in African American and Asian patients with prostate tumors harboring less TMPRSS2/ERG fusion.

Finally, miR-200b subfamily members and miR-205 are not induced by ERG in murine prostate derived from the pbsn-ERG transgenic mice, suggesting that ERG transgenic mice do not fully mimic the TMRPSS2/ERG-dependent prostate cancer development in human. It will be important to generate transgenic mice that overexpress these miRNAs in murine prostate luminal cells, and evaluate their physiological role in prostate tumorigenesis and progression.

In summary, our results indicate that miRNAs are important components of the ERG transcriptional network in human prostate cancer. Although induction of the tumor suppressive miR-200b subfamily of miRNA by oncogenic ERG appears to be counterintuitive, it is consistent with the slow-growing nature of the vast majority of primary prostate tumors.

## MATERIALS AND METHODS

### Cell culture and transient transfection

Human prostate cancer cell lines were obtained from the ATCC via the Tissue Culture Core at the Baylor College of Medicine, and cultured in appropriate media in a 5% CO2 incubator at 37°C. Specifically VCaP cells were cultured in DMEM high glucose (Life Technologies) supplemented with 10% FBS and 1 nM R1881. LAPC4 cells were cultured in IMDM (Life Technologies) with 10% FBS, 1 nM R1881, and 1× Glutamax (Thermo Fisher Scientific). LNCaP and 22RV1 cells cultured in RPMI1640 (Life Technologies) with 10% FBS. PC3 cells were cultured in DMEM/F12 (Life Technologies) with 10% FBS. DU145 cells were cultured in DMEM high glucose (Life Technologies) with 10% FBS. Cell cultures are discarded after 3 months or 15 passages and replenished from frozen stocks. Cells were regularly tested for mycoplasma contamination. Individual Silencer siRNAs against ERG were purchased from Thermo Fisher Scientific. 50 nM of siRNA in Opti-MEM were transiently transfected into cells using Lipofectamine RNAiMAX (Thermo Fisher Scientific), following the reverse transfection protocol. Cells were harvested four days after transfection for real time qPCR analyses. To measure the expression level of miR-200b/a/429 primary transcript after ERG knockdown, VCaP cells were transiently transfected with 50 nM of ERG siRNAs. Cells were harvested three days later for RNA purification. Total RNA was reverse transcribed using high capacity RNA-to-cDNA kit (ThermoFisher, cat. No. 4387406) and primary transcript of miR-200b/a/429 was determined by real-time qPCR using TaqMan Pri-miRNA assay and normalized against total RNA (ThermoFisher, cat. No. Hs03303027_pri).

### Plasmid

To construct a lentiviral vector for the simultaneous expression of miR-200b, miR-200a and miR-429 in mammalian cells, 2310 bp of human miR-200b/a/429 genomic DNA was amplified by PCR and cloned into EcoRI/BamHI site of the pCDH-CMV-MCS-Puro vector (System Biosciences, Mountain View, CA, USA), resulting in pCD-HmiR-200b/a/429. The sequences of two PCR primers are: 5′-CCA GGT GAA TTC CAG GAC CCA AAG CTG GTG-3′, and 5′-ACT GGC GGA TCC GAG GGT GGG GCA CAA GAG-3′. To construct a lentiviral vector for the expression of miR-205 in mammalian cells, 410 bp of human miR-205 genomic DNA was amplified by PCR and cloned into EcoRI/BamHI site of the pCDH-CMV-MCS-Puro, resulting in pCDH-miR-205. The sequences of two PCR primers are: 5′-CCA GGT GAA TTC TCT CCC AAA TGT GTG ATT CC-3′; and 5′-CCA TCT GGA TCC CTT TTT CCA ATG TGC CCA TC-3′. To express all four miRNAs, 410 bp of human miR-205 genomic DNA was amplified by PCR and cloned into BamHI/NotI site of the pCDH-miR-200b/a/429 vector, resulting in pCDH-miR-200b/a/429/205. The sequences of PCR primers are: 5′-CCA GGT GGA TCC TCT CCC AAA TGT GTG ATT CC-3′; and 5′-CCA TCT GCG GCC GCT TTT TCC AAT CTG CCC ATC-3′. 168 bp of miR-200b/a/429 promoter region that contains ETS-1 and ETS-2 was amplified by PCR and cloned into Acc65I/XhoI site of pGL3-Basic vector (Promega). The sequences of PCR primers are: 5′-GTC ACT GGT ACC TCG AAA CTG TCC CAG AGA CG-3′; and 5′-CGT TCC CTC GAG CTG GGT GCT CTG CCT CAG-3′. The site-directed mutagenesis was performed by double PCR strategy. The PCR primers to mutate ETS-1 site are: 5′-CAG GTC TGA ACT GAC CCT GTG CCA GGG CCT-3′; and 5′-AGG CCC TGG CAC AGG GTC AGT TCA GAC CTG-3′. The PCR primers to mutate ETS-2 site are: 5′-GCC TGA GCG GGG GCA AAG CTC ACC CTT GCA-3′; and 5′-TGC AAG GGT GAG CTT TGC CCC CGC TCA GGC-3′.

### Quantitative real time PCR (qPCR)

miRNA levels were determined by SYBR RT-qPCR. The first strand was synthesized using Mir-X^™^ miRNA First-Strand Synthesis Kit (Clontech, Palo Alto, CA, USA) according to the manufacturer's instructions. The sequences of miRNA-specific qPCR primers are as follows: 5′-TAA TAC TGC CTG GTA ATG ATG A-3 (miR-200b); 5′-TAA CAC TGT CTG GTA ACG ATG-3′(miR200a); 5′-TAA TAC TGT CTG GTA AAA CCG T-3′ (hsa-miR-429); 5′-TAA TAC TGT CTG GTA ATG CCG T-3′ (mmu-miR-429); 5′-TCC TTC ATT CCA CCG GAG T-3′ (miR-205). RNU48 was used as an internal control for comparing miRNA expression levels across different PCa cell lines. U6 snRNA served as an internal control for PC3 stable cell lines that express exogenous miRNAs. Reverse transcribed miRNAs were analyzed by real-time PCR using SensiFAST SYBR green (Bioline).

### Western blot analysis and antibodies

Rabbit anti-ERG monoclonal antibody (EPR3864, Abcam Inc. Cambridge, MA) and mouse anti-GAPDH monoclonal antibody (sc-32233, Santa Cruz Biotechnology) were used for western blot analysis. Rabbit anti-ERG polyclonal antibody (sc-354, Santa Cruz Biotechnology) was used for Chromatin Immunoprecipitation (ChIP) assay. Western blot analysis was using standard procedures.

### ChIP assay

VCaP cells were cultured in DMEM high glucose media supplemented with 10% FBS and 1 nM R1881. The ChIP-IT Express kit (Active Motif) was used to perform the assay following the manufacturer's protocol. Briefly, 1.5 × 10^7^ cells were cross-linked in fixation solution and lysed to release the nuclei. Chromatin released from the nuclei was sonicated. The supernatant containing the sheared chromatin was used in immunoprecipitation. 5 μg of anti-ERG rabbit polyclonal antibody (sc-354, Santa Cruz Biotechnology) or control normal rabbit IgG (sc-2027, Santa Cruz Biotechnology) were added to chromatin and incubated overnight at 4°C. The immune complexes were collected using protein A-agarose beads, followed by extensive washing. The chromatin DNA-protein-antibody complexes were eluted and DNA-protein formaldehyde cross-links were reversed. The DNA fragments were purified by using a QIAquick PCR purification kit (QIAGEN) and analyzed by real-time PCR using SensiFAST SYBR green (Bioline). The PCR primers used for amplification of ERG binding region of miR-200b/a/429 promoter are: 5′-CCA CCT GTG CAG GTC TGA -3′and 5′-CTG CAA GGG TGA GCT TCC-3′.

### Cancer cell proliferation and invasion assay

Cell proliferation was measured by using MTS assay. Briefly, PC3 stable cells were seeded at a density of 4 × 10^3^ cells per well in flat-bottomed 96-well plates (day 0) and their growth was measured on days 3. Cell media were changed once on day 2. CellTiter 96 Aqueous One Solution Reagent (Promega) was added to each well. After 1 hr incubation, the cell viability was measured by determining the absorbance at 490 nM using the Multiskan FC microplate photometer (Thermo Scientific). The Matrigel invasion assay was performed using the BD Biocoat Matrigel Invasion Chamber (BD Biosciences) following the manufacturer's instructions, and the pictures were taken using the EVOS XL Cell Imaging system (ThermoFisher).

### Transgenic mice

The prostate-specific ERG overexpression transgenic mice (Pbsn-ERG) were purchased from Jackson Laboratories, strain name: STOCK Tg(Pbsn-ERG*)1Vv/J, and was previously reported [42]. The mice were housed in a temperature-controlled animal facility at Baylor College of Medicine with a 12-hr light, 12-hr dark photocycle and provided water and rodent chow meal *ad libitum*.

### Statistical analysis

Student's *t*-test was used for statistical analyses. A *p*-value cutoff of 0.05 was used to determine significance.
